# Construct a nomogram prediction and evaluation of influencing factors of adverse pregnancy outcomes in GDM patients based on plasma miR-144-3p levels

**DOI:** 10.3389/fendo.2025.1548780

**Published:** 2025-06-23

**Authors:** Sanqiang Niu, Pengwen Wu, Bing Li, Huaili Xin, Kangjun Yu, Ningning Du

**Affiliations:** ^1^ Department of Obstetrics and Gynecology, The People’s Hospital of Bozhou, Bozhou, Anhui, China; ^2^ Department of Obstetrics and Gynecology, Bozhou Hospital of Anhui Medical University, Bozhou, Anhui, China; ^3^ Graduate School, Bengbu Medical University, Anhui, Bengbu, China; ^4^ Department of Reproductive Medicine, Bozhou People’s Hospital, Bozhou, Anhui, China

**Keywords:** gestational diabetes mellitus, miR-144-3p, adverse pregnancy outcomes, nomogram, influencing factors

## Abstract

**Objective:**

To examine the expression levels of miR-144-3p in the plasma of patients with gestational diabetes mellitus (GDM) and to construct a nomogram for predicting and evaluating factors influencing adverse pregnancy outcomes (APO) in GDM based on plasma miR-144-3p levels.

**Methods:**

This study included 442 pregnant women, comprising 216 diagnosed with GDM (GDM group) and 226 with normal glucose tolerance (NGT group). Plasma miR-144-3p levels in both groups were measured using reverse transcription real-time polymerase chain reaction (RT-qPCR). The diagnostic performance of plasma miR-144-3p for GDM was assessed by receiver operating characteristic (ROC) curve analysis. During pregnancy, the GDM group was followed, and outcomes were categorized into two groups: 132 with favorable pregnancy outcomes (FPO) and 84 with APO. A random number table method was applied to divide the GDM group into a training set (n=151) and a validation set (n=65) using a 7:3 ratio. Differences in variables across pregnancy outcome subgroups in the training set were examined. Univariate and multivariate logistic regression analyses were performed to identify risk factors for APO in GDM. Based on these factors, a nomogram prediction model was developed to estimate the risk of APO in GDM. The model’s performance was evaluated using area under the curve (AUC) analysis, calibration curve analysis, and decision curve analysis (DCA).

**Results:**

The expression of miR-144-3p was significantly higher in the GDM group than in the NGT group (*p* < 0.05). miR-144-3p showed an AUC of 0.877, with a sensitivity of 81.09% and a specificity of 91.20% for diagnosing GDM. No statistically significant differences were observed in general clinical characteristics between the training and validation sets. In the training set, gestational weight gain (GWG), the number of OGTT abnormalities, glycaemic control (GC), and miR-144-3p expression varied significantly between the APO and FPO subgroups (*p* < 0.05). Multivariate logistic regression analysis identified increased GWG, the number of OGTT abnormalities, poor GC, and higher miR-144-3p levels as independent risk factors for APO in GDM. The AUC of the nomogram based on these variables was 0.881 in the training set and 0.855 in the validation set. Calibration curves indicated good agreement between predicted and actual outcomes in both sets. The DCA showed a clear net clinical benefit and stable predictive utility.

**Conclusion:**

Elevated plasma miR-144-3p levels in pregnant women with GDM may contribute to the occurrence of APO. The number of OGTT abnormalities and glycaemic control were also identified as independent risk factors. A nomogram incorporating miR-144-3p and these clinical indicators displays strong predictive accuracy and provides a practical tool for assessing APO risk in GDM.

## Introduction

Gestational diabetes mellitus (GDM) is defined as the onset of diabetes mellitus during pregnancy, arising from abnormalities in maternal glucose metabolism. It is a relatively common complication associated with high-risk pregnancies. GDM affects an estimated 1.1–14.3% of pregnant women, with a recurrence rate between 35% and 70% (1). The condition involves disruptions in both glucose and lipid metabolism, posing substantial risks to mothers, fetuses, and newborns. As such, it has become a public health issue that threatens maternal and infant health (2). Current treatment strategies include dietary regulation, structured physical activity, and pharmacological approaches such as insulin therapy. However, despite these interventions, adverse pregnancy outcomes still occur in some patients (3). Identifying the risk factors associated with these outcomes in GDM is therefore essential to inform early clinical interventions aimed at improving maternal and neonatal prognosis.

MicroRNA (miRNA) is a class of small non-coding RNAs, approximately 22 nucleotides long, that controls gene expression by degrading or suppressing the translation of messenger RNA (4). It is involved in various biological processes, including diabetes and GDM (5). To date, over 2,500 miRNAs have been identified in humans, collectively regulating around 60% of the genome’s genes (6). Emerging studies have drawn attention to the role of circulating miRNAs in intercellular signaling, indicating that extracellular miRNAs may influence physiological activities (7). In recent years, miRNAs have been recognized as central players in metabolic regulation during pregnancy, influencing essential physiological processes and potentially serving as indicators of both maternal condition and fetal development (8). Increasing evidence links maternal miRNAs to pregnancy complications such as placental abruption, placenta previa, preeclampsia, gestational hypertension, fetal growth restriction, macrosomia, and GDM (9, 10). As such, miRNA profiling may shed light on the mechanisms contributing to GDM. Research has suggested a connection between miRNAs and insulin secretion and resistance, two major components in diabetes development and management (4). Elevated levels of miR-144 have been reported in the peripheral blood of pregnant women with GDM and in individuals with type 1 and type 2 diabetes (11). miR-144-3p and miR-144-5p are mature strands of miR-144, with miR-144-3p being particularly abundant in the peripheral blood of women with GDM (12). However, the specific relationship between miR-144-3p and GDM, along with its possible involvement in the development of GDM complicated by adverse pregnancy outcomes (APO), remains uncertain.

Given this background, the present study aimed to explore the association between plasma miR-144-3p levels and adverse pregnancy outcomes in women with GDM. Additionally, the study sought to build a predictive model for APO risk in this population, offering new perspectives for the clinical management and prevention of APO in GDM.

## 2 Materials and methods

### 2.1 Data collection

**Figure 1:**
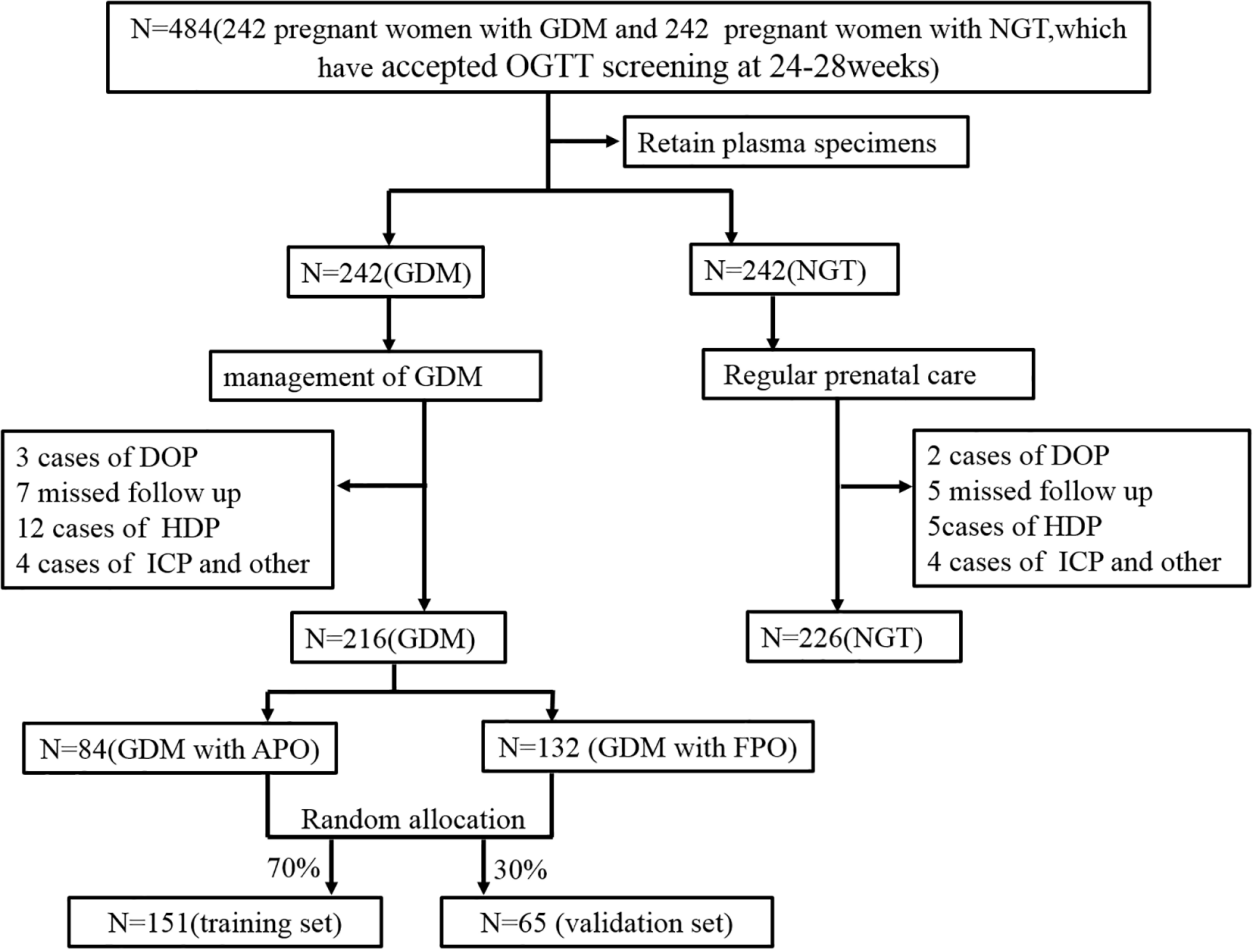
Flow chart of the study. APO, adverse pregnancy outcomes; FPO, favorable pregnancy outcomes; OGTT, Oral Glucose Tolerance Test; GDM, gestational diabetes mellitus; NGT, normal glucose tolerance; DOP, Delivery in other hospitals; HDP, hypertensive disorders of pregnancy; ICP, intrahepatic cholestasis of pregnancy.

A total of 484 pregnant women who received routine prenatal care at The Affiliated Bozhou Hospital of Anhui Medical University between June 2023 and June 2024 were initially assessed (all underwent OGTT screening at 24–28 weeks). GDM was diagnosed according to IADPSG criteria: fasting blood glucose ≥ 5.1 mmol/L; 1-hour blood glucose after a 75 g OGTT ≥ 10.0 mmol/L; 2-hour blood glucose after a 75 g OGTT ≥ 8.5 mmol/L (13). After applying inclusion and exclusion criteria, 442 participants were enrolled, excluding those with incomplete data. Of these, 216 were diagnosed with GDM (GDM group), while 226 had normal glucose tolerance (NGT group). The GDM group was followed through pregnancy, and outcomes were divided into a FPO group with 132 cases, and an APO group with 84 cases. A random number table method was used to divide the GDM group into a training set (n=151) and a validation set (n=65) at a 7:3 ratio.Inclusion criteria: (1) both groups met the GDM diagnostic criteria and received routine treatment upon admission; (2) all pregnancies were natural, with gestational age over 24 weeks, and were singletons; (3) voluntary participation in the study; (4) good patient compliance and ability to complete follow-up.Exclusion criteria: (1) other pregnancy complications such as gestational hypertension or intrahepatic cholestasis; (2) cardiopulmonary, hepatic, or renal dysfunction; (3) pre-existing diabetes, thyroid disease, adrenal disorders, or other endocrine conditions; malignancies; (4) hematologic or autoimmune diseases; (5) psychiatric illnesses; (6) unhealthy lifestyle habits, including alcohol use and smoking. All participants provided written informed consent and were cooperative throughout the study period. The screening flowchart is shown in (**Figure 1**). This study was conducted in accordance with the ethical standards of the World Medical Association Declaration of Helsinki and relevant clinical research regulations. Approval was obtained from the academic ethics committee of The Affiliated Bozhou Hospital of Anhui Medical University.

### 2.2 Blood specimen collection and blood glucose testing

A 4 mL sample of peripheral venous blood was collected into EDTA tubes from participants in the GDM group prior to their initial diagnosis and before receiving any hypoglycemic treatment. For the NGT group, samples were obtained during routine prenatal visits. All samples were centrifuged at 3000 r/min for 10 minutes to separate the plasma, which was then transferred to nuclease-free EP tubes and stored at -80°C. Following the protocol for oral glucose tolerance testing (OGTT), all participants fasted for at least 8 hours before the test and consumed 300 mL of a 75 g glucose solution within 5 minutes. Venous blood samples were collected before ingestion, as well as 1 hour and 2 hours after glucose intake, to assess glucose levels, including fasting and postprandial glucose. Plasma glucose concentrations at the 1-hour and 2-hour marks were determined using the glucose oxidase method.

### 2.3 Plasma RNA acquisition and RT-qPCR assay

Total RNA was extracted from 500 μL of plasma using TRIzol LS reagent (Vazyme, Nanjing, China) following the manufacturer’s instructions. RNA concentrations were measured using a NanoDrop One Spectrophotometer (Thermo Fisher Scientific), and sample purity was assessed with a 2100 Bioanalyzer (Agilent). cDNA was synthesized from 2 μL of RNA using the miRNA 1st Strand cDNA Synthesis Kit (Vazyme, Nanjing, China). miR-144-3p expression was then measured using miRNA Universal SYBR Green qPCR Master Mix (Vazyme, Nanjing, China) according to the manufacturer’s protocol. Thermal cycling conditions were: 5 minutes at 94°C, 10 seconds at 95°C, and 30 seconds at 60°C, repeated for 40 cycles. U6 served as the internal control. Relative expression levels of miR-144-3p were calculated using the 2^-ΔΔCt^ method, normalized to U6 small RNA. The primer sequences were as follows: miR-144-3p RT primer: 5’-GTCGTATCCAGTGCAGGGTCCGAGGTATTCGCACT GGATACGACAGTAC A-3’;miR-144-3p upstream primer sequence was 5’- GCGCGCGTACAGTATAGAT GA-3’ and downstream primer sequence was 5’- AGTGCAGGGTCCGAGGTATT-3’; U6 upstream primer sequence was 5’-CTCGCTTCGGCAGCAC-3’ and downstream primer sequence was 5’-AACGCTTCACGAATTTGCGT-3’.

### 2.4 Intervention methods

Pregnant women diagnosed with GDM were monitored for blood glucose levels throughout the course of pregnancy. Dietary regulation and physical activity guidance were provided by the Obstetrics and Nutrition Clinic. For those with poor glycaemic control, referrals were made to the endocrinology department for insulin treatment. All participants received health education, along with dietary and exercise instructions. When necessary, medications such as metformin and insulin were administered. Follow-up visits were scheduled regularly at the outpatient clinic until delivery.

### 2.5 Determination of maternal and perinatal pregnancy outcomes

Pregnant women with coexisting conditions such as gestational hypertension and intrahepatic cholestasis were excluded, along with those presenting with less common complications. All participants were monitored until delivery. Maternal and neonatal outcomes were recorded and analyzed for both groups. Maternal adverse outcomes included preterm rupture of membranes, genitourinary tract infections, threatened preterm labor, postpartum hemorrhage, and postpartum infections. Perinatal adverse outcomes included preterm birth, macrosomia, fetal distress, neonatal asphyxia, neonatal hyperbilirubinemia, and neonatal hypoglycemia. The objective was to compare the rates of adverse maternal and neonatal outcomes between the experimental and control groups.

### 2.6 Construction and validating nomogram model for GDM pregnant women with APO

Variables that showed significant associations in univariate analysis were further assessed using multivariate logistic regression. Predictors that remained significant were selected as risk factors for APO in pregnant women with GDM, and these were used to construct the nomogram model. The AUC was applied to evaluate the model’s discriminative capacity. DCA was conducted to assess its clinical applicability. Calibration curves were generated using the Bootstrap method (B=1000), ensuring that the nomogram model is robust, consistent, and practically useful for predicting APO in GDM pregnancies.

### 2.7 Statistical analysis

All statistical analyses were conducted using SPSS version 25.0. For normally distributed continuous variables, data were presented as mean ± standard deviation and analyzed using Student’s *t* test. Categorical variables were expressed as percentages and compared using the chi-square test. Non-normally distributed variables were reported as M (P25~P75) and analyzed using the Mann-Whitney U test. The diagnostic value of plasma miR-144-3p for GDM was assessed using ROC curve analysis. Univariate and multivariate logistic regression analyses were conducted to identify factors linked to APO in GDM. The nomogram model was developed using the “rms” package in R based on the variables identified as significant. Predictive performance was assessed via ROC curves. Internal validation of the nomogram model was conducted with the Bootstrap method, and calibration curves were generated accordingly. DCA curves were plotted using the “rmda” package in R. A *p* < 0.05 was considered statistically significant.

## 3 Results

### 3.1 Baseline characteristics of the participants

Of the 442 pregnant women who met the inclusion criteria, 216 were diagnosed with GDM, while 226 had NGT and were included in the control group. Comparative analysis showed no significant differences between the two groups in terms of age, gravidity, parity, gestational week at blood collection, family history of diabetes, history of adverse pregnancy, systolic and diastolic blood pressure, pre-pregnancy body mass index, and gestational weight gain, as shown in **Table 1**.

**Table 1:** Comparison of baseline characteristics between GDM group and the NGT group.

Variable	GDM group (n=216)	NGT group (n=226)	*t/χ^2^/Z*	*P value*
Age (years)	28.88±3.82	28.59±3.78	0.802	0.423
Gravidity, M (P25-P75)	2 (1~2)	2 (1~2)	0.545^a^	0.586
Parity, M (P25-P75)	1 (1~2)	1 (1~2)	0.008 ^a^	0.944
FHD, n (%)			0.071^b^	0.789
yes	13	15		
no	203	211		
AMH, n (%)			0.106^b^	0.745
yes	12	11		
no	204	215		
BTG(wk)	27.84±0.91	28.02±1.19	1.750	0.081
SBP (mmHg)	109.50 ± 8.73	108.51 ± 8.76	1.139	0.255
DBP (mmHg)	72.86 ± 8.56	71.74 ± 8.29	1.389	0.166
Pre-BMI (kg/m^2^)	23.20±1.61	23.04±1.68	1.064	0.288
GWG (kg)	14.13±2.62	13.72 ± 2.59	1.630	0.104

GDM, Gestational diabetes mellitus; NGT, Normal glucose tolerance; FHD, Family history of diabetes; AMH, Adverse maternity history; BTG, Blood test for gestational age; SBP, Systolic blood pressure; DBP, Diastolic blood pressure; pre-BMI, Pre-pregnancy body mass index; GWG, Gestational weight gain; OGTT, Oral glucose tolerance test; a: Mann-Whitney U-test results; b: *χ^2^ -test* esults.

### 3.2 MiR-144-3p expression and diagnostic value in GDM

**Figure 2:**
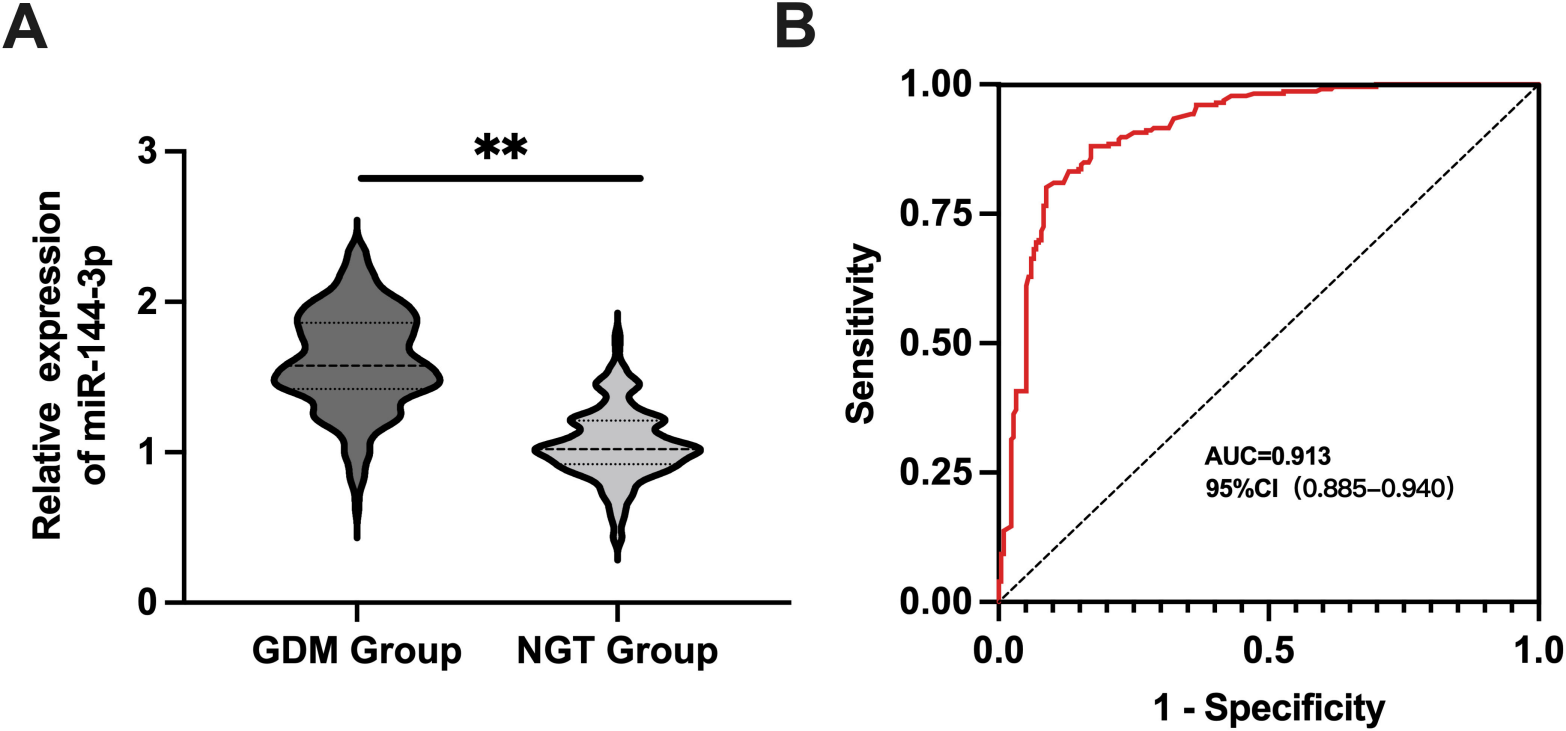
MiR-144-3p expression and diagnostic value in GDM. **(A)** The expression level of miR-144-3p in the plasma of pregnant women with GDM group and NGT group. **(B)** The ROC curve detected the diagnostic value of miR-144-3p in GDM. **P <0.05.

Plasma levels of miR-144-3p were significantly higher in the GDM group compared to the NGT group (*p* < 0.05), as shown in **Figure 2A**. ROC analysis revealed an AUC of 0.913 for miR-144-3p, with a cutoff value of 1.222 and a 95% CI ranging from 0.885 to 0.940. Sensitivity was 81.09%, and specificity was 91.20%, indicating strong diagnostic performance, as shown in **Figure 2B**.

### 3.3 Comparison of baseline characteristics between training set and validation set

No significant differences were observed in baseline characteristics between the training and validation sets, as summarized in **Table 2**.

**Table 2:** Comparison of basic characteristics between training set and validation set.

Variable	Training set (n=151)	Validation set (n=65)	*t/χ^2^/Z*	*P value*
Age (year)	28.87±3.98	28.91±3.44	0.070	0.944
Gravidity, M (P25-P75)	2 (1~2)	2 (1~2)	0.862 ^a^	0.427
Parity, M (P25-P75)	1 (1~2)	1 (1~2)	1.588^a^	0.112
FHD, n%			0.461^b^	0.497
yes	8	5		
no	143	60		
AMH, n (%)			0.003^b^	0.956
yes	9	4		
no	142	61		
BTG (wk)	27.81±0.86	27.92±1.03	0.811	0.418
SBP (mmHg)	110.78 ± 8.65	108.95 ± 8.73	1.422	0.156
DBP (mmHg)	71.63 ± 7.79	73.38 ± 8.85	1.453	0.147
Pre-BMI (kg/m^2^)	23.24±1.54	23.15±1.80	0.374	0.709
GWG (kg)	13.99±2.51	14.44 ± 2.86	1.158	0.248
Number of OGTT anomalies, M (P25-P75)	2 (1~2)	2 (1~2)	0.520^a^	0.603
GC, n%			0.178 ^b^	0.674
good	93 (61.6)	42 (64.6)		
bad	58 (38.4)	23 (35.4)		
MiR-144-3p	1.59±0.33	1.63 ± 0.33	0.817	0.415

GDM, Gestational diabetes mellitus; NGT, Normal glucose tolerance; FHD, Family history of diabetes; AMH, Adverse maternity history; BTG, Blood test for gestational age; SBP, Systolic blood pressure; DBP, Diastolic blood pressure; pre-BMI, Pre-pregnancy body mass index; GWG, Gestational weight gain; OGTT, Oral glucose tolerance test; GC, Glycaemia control; a: Mann-Whitney U-test results; b: *χ^2^ -test* esults.

### 3.4 Univariate and multivariate logistic regression analysis of the training set for APO

**Figure 3:**
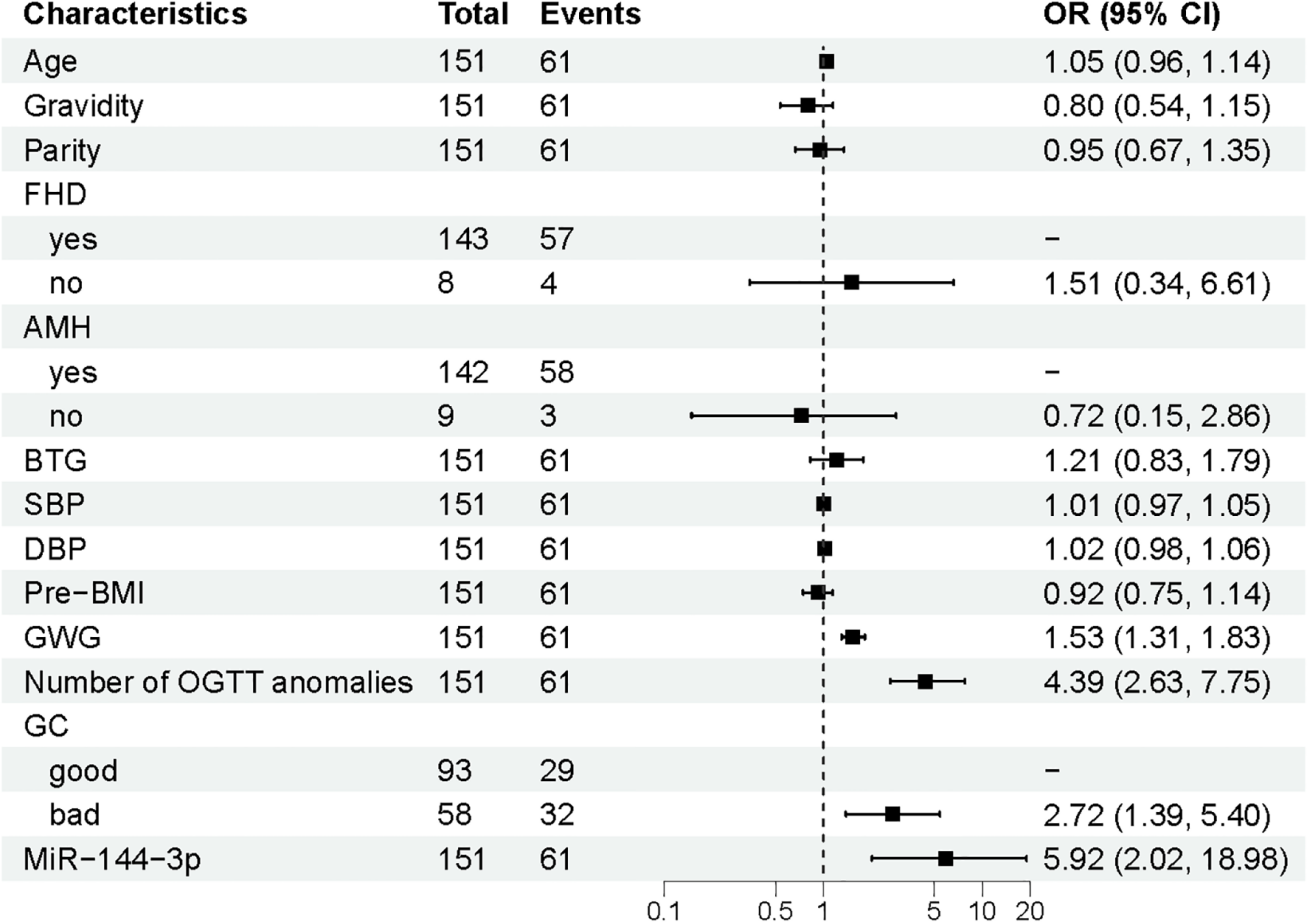
Multivariate logistic regression analysis of APO. APO, adverse pregnancy outcomes; GWG, Gestational weight gain; OGTT, Oral glucose tolerance test; GC, Glycaemia control; OR, odds ratio; CI, Confidence interval.

**Figure 4:**
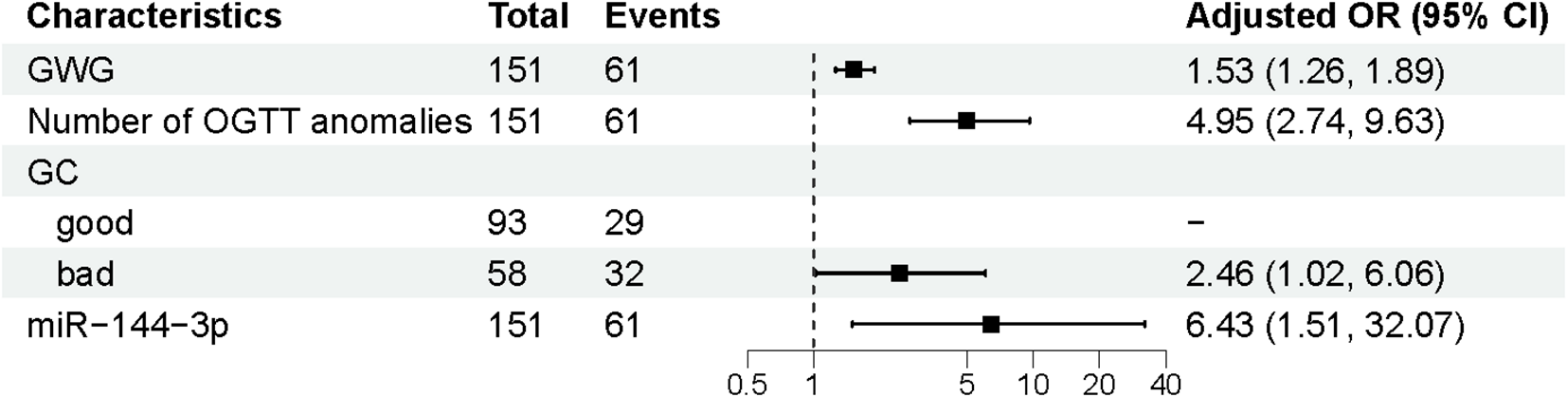
Univariate logistic regression analysis of APO. APO, adverse pregnancy outcomes; FHD, Family History of Diabetes; BTG, Blood test for gestational age; SBP, Systolic blood pressure; DBP, Diastolic blood pressure; pre-BMI, Pre-pregnancy body mass index; GWG, Gestational weight gain; OGTT, Oral glucose tolerance test; GC, Glycaemia control; OR, odds ratio; CI, Confidence interval.

Univariate logistic regression analysis conducted on the training set identified significant associations between APO and four variables: GWG, the number of OGTT abnormalities, glycaemic control, and miR-144-3p expression (*p* < 0.05) (**Figure 3**). These four factors were included in the final multivariate logistic regression model for risk prediction (**Figure 4**). The analysis confirmed that GWG, number of OGTT abnormalities, glycaemic control status, and miR-144-3p expression were independent risk factors for APO in GDM. A logistic regression equation was derived to quantify their contribution: GDM with APO =−12.601 + 0.422(GWG)+1.599(Number of OGTT anomalies) +0.899(GC_bad_) +1.862 (MiRNA-144-3p).

### 3.5 Construction of a nomogram model for GDM pregnant women with APO

**Figure 5:**
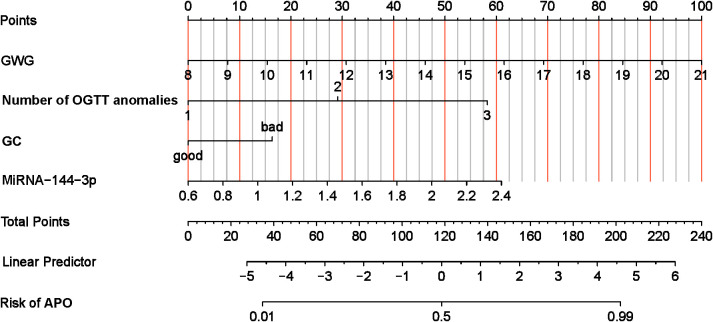
Nomogram of risk of GDM with APO. APO, adverse pregnancy outcomes; GWG, Gestational Weight Gain; GC, Glycaemia control; OGTT, oral glucose tolerance test.

Based on the results of the multivariate analysis, a nomogram model was established to predict the risk of APO in GDM patients (**Figure 5**). The model incorporated four independent risk variables: GWG, number of OGTT abnormalities, GC, and plasma miR-144-3p level.

### 3.6 Validating the nomogram predictive model

**Figure 6:**
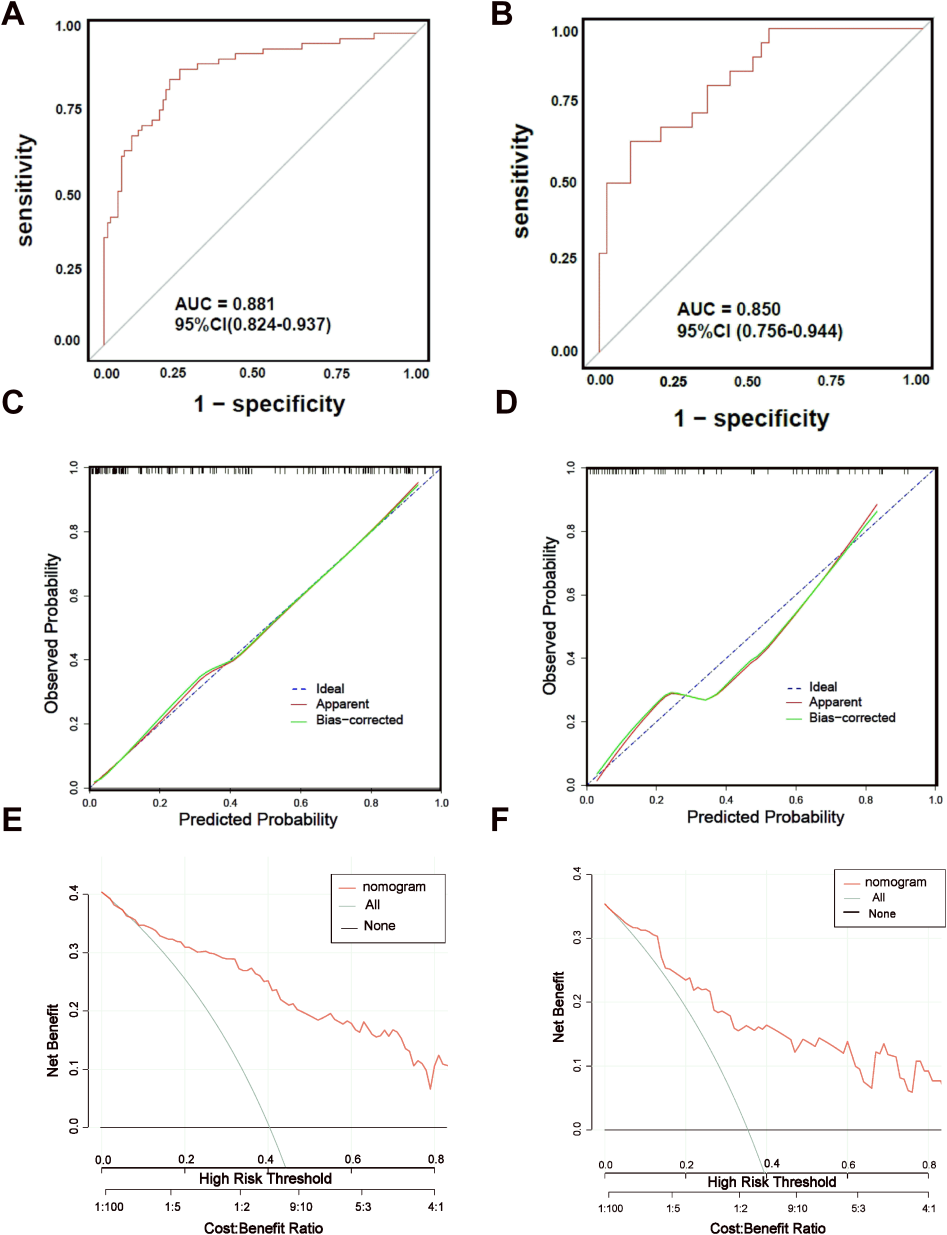
ROC curves, Calibration curves and DCA curves of nomogram model. **(A)** ROC curve of nomogram model of GDM with APO in training set. **(B)** ROC curve of nomogram model of GDM with APO in training set. **(C)** Calibration curve of nomogram model of GDM with APO in training set.**(D)** Calibration curve of nomogram model of GDM with APO in validation set. **(E)** DCA curve of nomogram model of GDM with APO in validation set. **(F)** DCA curve of nomogram model of GDM with APO in validation set.

The discriminative ability of the nomogram model was assessed through ROC curve analysis, applied to both the training and validation datasets. The AUC for the training set was 0.881 (95% CI: 0.824–0.937, *p* < 0.05), and for the validation set, it was 0.850 (95% CI: 0.756–0.944, *p* < 0.05), indicating strong predictive performance. Calibration curves, generated using the Bootstrap method (B=1000), showed good alignment between predicted probabilities and actual outcomes. DCA was used to assess the clinical usefulness of the nomogram, measuring net benefits against varying risk thresholds. The DCA results supported a high net benefit and solid clinical predictive value of the model (**Figure 6**).

## 3 Discussion

GDM is a major pregnancy-related complication that threatens the health of both mothers and their newborns (14). While many patients can maintain stable blood glucose levels through structured interventions such as diet, exercise, and medication, a subset still faces a higher likelihood of experiencing APO, even when glycaemic control appears adequate following treatment (15). In this study, the incidence of maternal and perinatal APO was found to be higher in the GDM group compared to the NGT group, which is consistent with the results reported by Moon et al. (16). These findings reinforce the idea that GDM significantly increases the risk of adverse outcomes for both mother and child. Therefore, identifying reliable biological markers capable of evaluating pregnancy outcomes in GDM is critical for guiding clinical efforts to reduce the occurrence of such complications.

Plasma miR-144-3p is a small RNA molecule located on human chromosome 17q11.2 and is recognized as a key microRNA (miR) with important regulatory functions in biological systems (17). Yan et al. reported significantly elevated levels of miR-144-3p in peripheral blood mononuclear cells from individuals with type 2 diabetes (18). In line with these results, our study showed that plasma miR-144-3p levels were higher in pregnant women with GDM compared to those with NGT, suggesting that increased miR-144-3p expression may be involved in the development and progression of diabetes. As recommended by the International Diabetes Association’s Pregnancy Group, a 75 g OGTT is performed between 24 and 28 weeks of gestation to diagnose GDM (19). Our study further validated the diagnostic utility of miR-144-3p through ROC analysis, indicating that elevated miR-144-3p levels may serve as an auxiliary diagnostic marker for GDM. Yu et al. reported that miR-144-3p promotes the production of reactive oxygen species in response to hyperglycemia, worsening diabetic cardiomyopathy (20). Similarly, Wei et al. found that miR-144-3p contributes to the progression of diabetic keratopathy by influencing autophagy and apoptosis (21). Additional studies have shown that miR-144-3p impacts insulin secretion and sensitivity (22), reinforcing its role in the development of diabetes and associated complications (20). While the specific mechanisms and regulatory pathways of miRNAs in GDM with APO are not yet fully understood, our findings show that miR-144-3p levels were higher in GDM patients who experienced APO than in those who did not, indicating that plasma miR-144-3p may play a part in the onset of APO in the context of GDM.

Abnormal glucose levels observed during the 75 g OGTT reflect both pancreatic beta cell dysfunction and increased insulin resistance. A greater number of abnormal OGTT values indicates delayed peak insulin secretion, more severe insulin resistance, and a tendency toward prolonged hyperglycemia in the postpartum period (23, 24). Zhou et al. showed that the different OGTT values capture distinct hyperglycemic profiles, each of which may influence the risk of pregnancy complications to varying degrees. Specifically, when GDM patients exhibit OGTT values exceeding 5.1, 10.0, or 8.5 mmol/L at fasting, 1 hour, and 2 hours respectively, there is a significant increase in the incidence of hypertensive disorders of pregnancy (HDP), preterm birth, neonatal hyperbilirubinemia, and macrosomia (25). In addition, excessive gestational weight gain (GWG) contributes to fluctuations in blood glucose, thereby increasing the likelihood of APO (26). A population-based cohort study in China involving 6.4 million participants found that poor glycaemic control in late pregnancy raised the risk of APO (27). In our study, women with GDM had significantly more abnormal OGTT values and higher GWG compared to those with NGT, suggesting that GDM is accompanied by more pronounced metabolic disruptions. Among GDM cases, those who experienced APO also had a higher frequency of abnormal OGTT results and greater GWG, reinforcing the association between these variables and the risk of adverse maternal and neonatal outcomes. This finding aligns with earlier reports (12). Moreover, elevated levels of circulating miR-330-3p have been linked to glycaemic control and APO in GDM (28), while reduced expression of serum miR-29a/b has been associated with pathological neonatal jaundice in GDM pregnancies (29). These findings support the notion that dysregulated microRNA expression may be involved in the development of neonatal complications, consistent with the observations from this study. In the present study, we further examined the factors influencing the occurrence of APO in GDM through multivariate logistic regression analysis and found that higher GWG, increased number of OGTT abnormalities, elevated miR-144-3p levels, and poor glycaemic control were all independent risk factors. Possible explanations include evidence from previous studies suggesting that prolonged hyperglycemia in pregnant women with GDM stimulates the release of inflammatory mediators, leading to damage of vascular endothelial cells (16). In the fetus, elevated glucose levels trigger oxidative stress by increasing oxygen free radicals, which can harm fetal tissues. In addition, raised fasting and postprandial glucose levels contribute to the overproduction of bile acids, potentially resulting in intrahepatic cholestasis. This condition may also stimulate the release of uterine prostaglandins, promoting uterine contractions and raising the risk of preterm labor. Other research has shown that excessive maternal glucose is transferred to the fetus via the placenta, increasing the likelihood of premature rupture of membranes (30, 31). Shen et al. (32) demonstrated that miR-144-3p promotes adipogenesis and raises serum total cholesterol levels in a mouse model. It is speculated that increased expression of miR-144-3p may be related to higher maternal weight gain during pregnancy, which could contribute to the secretion of various endocrine adipocytokines. This may intensify insulin resistance, suppress lipolysis, disrupt adipose protein synthesis, and worsen metabolic imbalances, ultimately increasing the risk of adverse maternal and neonatal outcomes.

To improve the accuracy of predicting the risk of GDM with APO, this study developed a nomogram model that serves as a straightforward and visual predictive tool. Calibration and ROC curve analyses of both the training and validation sets showed that the model achieved high accuracy in forecasting GDM with APO. Furthermore, DCA indicated that the nomogram offered a higher net benefit, highlighting its clinical value and practical utility. These findings suggest that the nomogram model has meaningful potential for clinical application and may support improved management of GDM complicated by APO.

In conclusion, miR-144-3p expression is elevated in the peripheral plasma of pregnant women with GDM and may be involved in the onset and development of GDM with APO. The predictive model that incorporates miR-144-3p and other independent risk factors shows strong predictive value for assessing the risk of APO in GDM. However, this study has certain limitations, primarily the exclusion of cases where GDM was accompanied by gestational hypertension or intrahepatic cholestasis. This decision aimed to reduce potential confounding effects from those comorbidities. Despite this limitation, the findings presented here offer valuable insights into this area and may contribute to lowering the incidence of APO. The model holds clear clinical relevance for diagnosis and risk assessment in GDM.

## Data Availability

The datasets presented in this study can be found in online repositories. The names of the repository/repositories and accession number(s) can be found in the article/**Supplementary Material**.
